# Extraction of specific parameters for skin tumour classification

**DOI:** 10.1080/03091900802451315

**Published:** 2009-04-21

**Authors:** M. Messadi, A. Bessaid, A. Taleb-Ahmed

**Affiliations:** † Biomedical Engineering Laboratory, Department of Biomedical Electronics, Sciences Engineering Faculty, Abou Bekr Belkaid University, 22 Rue Abi Ayad Abdelkrim, Fg Pasteur BP 119, Tlemcen 13000, Algeria; ‡ LAMIH UMR CNRS 8530 Laboratory, Valenciennes, le Mont Houy, France

**Keywords:** Segmentation, Pre-processing, Karhunen–Loeve transform, Fractals analysis, Melanoma recognition, Neural networks

## Abstract

In this paper, a methodological approach to the classification of tumour skin lesions in dermoscopy images is presented. Melanomas are the most malignant skin tumours. They grow in melanocytes, the cells responsible for pigmentation. This type of cancer is increasing rapidly; its related mortality rate is increasing more modestly, and inversely proportional to the thickness of the tumour. The mortality rate can be decreased by earlier detection of suspicious lesions and better prevention. Using skin tumour features such as colour, symmetry and border regularity, an attempt is made to determine if the skin tumour is a melanoma or a benign tumour. In this work, we are interested in extracting specific attributes which can be used for computer-aided diagnosis of melanoma, especially among general practitioners. In the first step, we eliminate surrounding hair in order to eliminate the residual noise. In the second step, an automatic segmentation is applied to the image of the skin tumour. This method reduces a colour image into an intensity image and approximately segments the image by intensity thresholding. Then, it refines the segmentation using the image edges, which are used to localize the boundary in that area of the skin. This step is essential to characterize the shape of the lesion and also to locate the tumour for analysis. Then, a sequences of transformations is applied to the image to measure a set of attributes (A: asymmetry, B: border, C: colour and D: diameter) which contain sufficient information to differentiate a melanoma from benign lesions. Finally, the various signs of specific lesion (ABCD) are provided to an artificial neural network to differentiate between malignant tumours and benign lesions.

## 1. Introduction

Melanoma has become one of the most dangerous diseases, and is seen in all the regions of the world. Its frequency is rising in many countries, for example, 10 cases were reported in each year in Algeria [1]. Currently, experienced dermatologists can identify a melanoma with 75% accuracy [2]. In this work, we are motivated by the desire to classify skin lesions as malignant or benign from colour photographic slides of the lesions. We used the ABCD rule (A: asymmetry, B: border, C: colour and D: diameter) to help distinguish between these different tumours. The choice of this rule is based on dermatology criteria: shape, colour and symmetry. The ABCD parameter has stimulated interest in adjunctive diagnostic modalities that might facilitate clinical recognition of melanoma, including the automated interpretation of colour images with computerized image analysis. Thus, there has been increasing interest in computer-aided systems for the clinical diagnosis of melanoma as a support for dermatologists in different analysis steps, such as lesion boundary detection, extraction of the ABCD parameters and classification into different types of lesions. The methodology we developed relies on extracting specific information attributes to be encrypted and then by setting a system combining the following modules:

- filtering (pre-processing);
- segmentation;
- extraction of ABCD attributes; and
- classification.

In order to better discriminate the different specific signs of lesions (ABCD), an image database is required to classify overall lesions; these steps are addressed in the next sections.

## 2. Pre-processing

**Figure 1:**
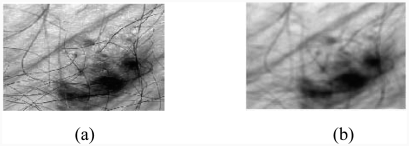
Median filtering: (a) original image, (b) result after median filtering.

Dermatologists can achieve early detection of skin tumours by studying the medical history of the patient, and also by examining the edge, shape, texture and colour of the lesion. Before such an examination, it is necessary to start by pre-processing and segmenting the skin tumour image. Technical difficulties in image segmentation include variations of brightness, the presence of artefacts (e.g. hair) and variability of edges. The idea is that if there is a transaction on edge detection of a source noised image, we can locate other additional edges due to the presence of noise. Therefore, filtering the noised image is necessary. In our system, we applied median filtering to minimize the influence of small structures (like thin hairs) and to isolate islands of pixels (like small air bubbles) in the segmentation result. For images including thick hairs with colour hue similar to that of the lesion which was not removable by the median filter (figure 1(b)), a specific hair removal technique called DullRazor [3] is applied. The last pre-processing step in our system is the application of the Karhunen–Loève transform, which enhances the edge, making easier extraction of the lesion from the surrounding skin.

### 2.1. Median filtering

The algorithm of the median filter, which is a nonlinear filter, replaces each pixel by the median value of the neighbouring pixels. This filter is used on skin tumour images. It is noticed that noise is not completely eliminated and some residual noise depicting some hair traces remained (figure 1). Such noise can adversely affect the segmentation quality.

### 2.2. Thick hair removal

**Figure 2:**
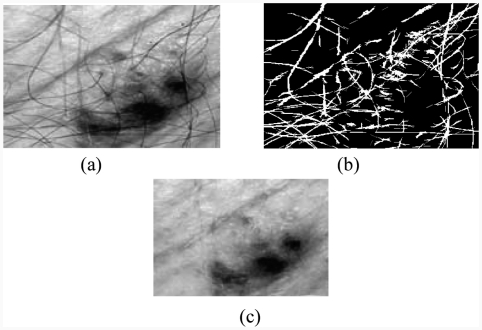
Hair removal by the DullRazor technique: (a) original image, (b) mask hairs, (c) image after removal of hair.

Some images include artefacts, mostly hair; these artefacts can be misleading for the segmentation algorithm. The DullRazor technique, an artefact removal pre-processing technique, deals well with hair and other artefacts. However, it tends to erase the details of the image by making the pigmented network unclear. We note from figure 2(c) that the results are much more interesting than those achieved by the median filter. The DullRazor algorithm [3] is as follows:

1. dilate then erode the image to remove the small details;
2. calculate the difference between the obtained image and the original one;
3. dilate then erode the mask of difference, to remove noise;
4. create a Boolean mask containing the location of the artefacts; and
5. from the original image, replace the pixels covered by the mask by the pixels corresponding to the original image.

### 2.3. Karhunen–Loève transform

The next pre-processing step consists of applying the Karhunen–Loève transform (KLT) to facilitate the segmentation process by enhancing the edges in the image. One of the most commonly methods used to achieve this reduction without losing too much information is principal component analysis (PCA), known as KLT in image processing. This method allows parameters to be extracted that reflect the distribution characteristics of the image. However, the recognition of complex textures (for example, cases of pigmented network) sometimes requires the spatial organization of pixels to be explicitly kept. It is possible to use traditional techniques in data compression, e.g. PCA, to reduce the information contained in the image, while preserving the structures to be detected. The purpose of KLT is to find a set of *M* orthogonal vectors in the data space that take better account of their variance [2]. The first vector is oriented along the axis corresponding to the maximum variation. The second vector is built in the subspace orthogonal to the first, inside this subspace; and is oriented in the direction of the maximum residual variance.

We reduce the dimensionality by projecting on a new base. This space generally contains most of the information in the original image.

(1) $$mx=\frac{1}{M}\sum_{k=1}^{M}X_{k}$$

(2) $$C_{x}=\frac{1}{M}\sum_{k=1}^{M}\left(X_{k}.X_{k}^{T}-m_{x}.m_{x}^{T}\right),$$

where *M* is the number of data, and *mx* is the average vector of the image.

We define a matrix *A* such that the lines are the eigenvectors of the matrix *C_x_* ordered by the decline in eigenvalues. The KLT of the vector X is defined by the following equation [4]:

(3) $$y=A.(X-m_{x}).$$

**Figure 3:**
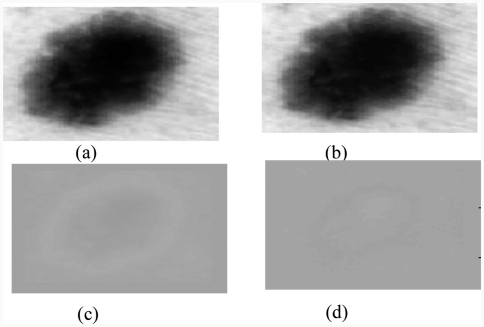
Results of the Karhunen–Loeve transform: (a) original image, (b) 1st principal component represents 98.87% of the total variance, (c) 2nd component represents 1.08%; (d) 3rd component represents 0.0036.

Due to the decreasing ordering of the eigenvalues and corresponding eigenvectors, the first principal component will contain the maximum variance. Since most variation occurs at edges between lesion and surrounding skin; the first principal component is a natural choice for segmentation (figure 3).

## 3. Segmentation

**Figure 4:**
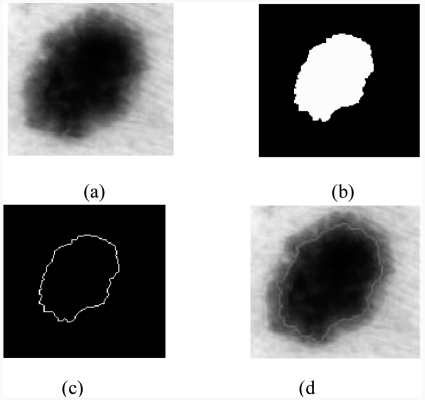
Result of the segmentation: (a) original image, (b) binary mask, (c) edge of the tumour, (d) segmented image.

The quality of interpretation of a colour image depends heavily on that of segmentation [5], which plays a major role in image processing and computer vision. It must achieve the difficult task of extracting useful information to locate and delineate regions present in the digital image [6]. This low level of processing allows the identification of the classes present in the image [12]. In order to provide a tool for segmentation, many methods have been developed and are based on the following steps:

1. The gradient is applied to an image to create a binary mask containing only the tumour, to calculate the gradient.
2. The mask binary gradient is dilated.
3. This leads to the final mask where more regions may contain the tumour. To obtain the tumour, the following steps are followed:
  - the hole regions are filled in;
  - regions touching the edge of the image are deleted; and
  - small regions with the same element are removed.
4. The largest region among the remaining regions is kept.
5. The outline of the tumour is drawn on the original image (figure 4).

## 4. Extraction of attributes

This work aims to design robust parameters that describe lesions, to ensure that melanoma and benign lesions can be distinguished. We used the ABCD rule to help distinguish between these different tumours [7]. This rule was chosen based on dermatology criteria: shape of the tumour, colour and symmetry. Based on the literature, we find that the list of attributes commonly used for automatic classification of lesions is linked to the ABCD rule. These criteria seek to capture information on:

- Asymmetry (A). According to dermatologists, melanomas develop in an anarchic fashion (i.e. they are asymmetrical), while benign tumours are symmetrical.
- Border irregularity (B): benign lesions are generally defined by clear boundaries; while melanomas are defined by much contrasted borders irregularly.
- Diameter (D). Melanomas usually start with a diameter of more than 6–7 mm.
- Colour (C). Melanomas are represented by several colours. The pigmentation of a lesion can be characterized by several colours—five to six colours may be present in a malignant lesion.

ABCD rules are commonly used by dermatologists. Yet a diagnosis made by a dermatologist based on the visual and quantitative evaluation of such criteria may be subjective. Thus, our main purpose is to characterize the ABCD criteria used as input to an automate classifier. However, each attribute alone is not sufficient to diagnose a lesion precisely. In other words, a combination of these attributes is necessary for diagnostic decision.

### 4.1. Asymmetry index

Asymmetry (A) is an essential parameter in differentiating malignant tumours from benign lesions. It is generally evaluated by dermatologists through observation by comparing the two halves of the lesion according to the principal axis. Stoecker *et al*. [8] has developed an algorithm to calculate an index of asymmetry. It uses the principal axes of the lesion: for a symmetrical lesion they are consistent with the symmetry axes. An index is calculated from the smallest difference between the image area of the lesion and the image of the lesion reflected from the principal axis. This value is reported in the area of the lesion, which allows for a percentage of asymmetry. Another method [9] calculates the index of asymmetry by the differences between the areas defined by the 180 axes (compared to the centre of gravity of the lesion). We can therefore conclude that asymmetry is a quantifiable property. Therefore, the asymmetry parameter can be used for discriminating and characterizing the melanomas. According to dermatologists four axes are sufficient to determine the rate of symmetry (vertical, horizontal and two diagonal axes) [8].

#### 4.1.1. Axis search of axial symmetry

The asymmetry is quantified related to the local origin of the lesion (L). The lesion is described by a binary image (*z*(*i*,*j*) = 1 if (*i*,*j*) ∈ *L*, 0 otherwise). The central symmetry can be determined by a rotation of 180° around the centre of gravity. In this paragraph, the axial symmetry around the principal and the secondary axis of inertia are considered.

**Figure 5:**
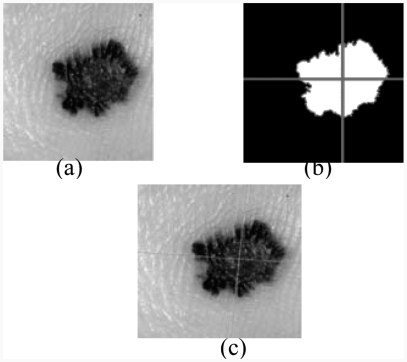
Calculating the symmetry following the two principal axes: (a) image after filtering, (b) binary mask, (c) detection axis of inertia.

To determine the principal and secondary axis of inertia, we consider the space (*o*, *x*, *y*) which represents all points of the lesion. The first axis represents the maximum variation, which is similar to the first moment of inertia (figure 5(c)). The second axis is orthogonal to the first one [2].

(4) $$I(\varphi )=\sum_{\left(i,j\right)}D_{\varphi }^{2}(i,j)=\sum_{\left(i,j\right)}{\left[-i\text{sin}(\phi )+j\text{cos}(\phi )\right]}^{2},$$

where *D*(*i*, *j*) is the distance between pixels (*i*, *j*) and its projection in the horizontal Cartesian axis. The longitudinal direction of a lesion is obtained by the derivation of the equation (4) which is given by the following equation [10]:

(5) $$\frac{\partial I(\varphi )}{\partial \varphi }=0\Leftrightarrow \varphi_{0}=0.5*{\text{tan}}^{-1}\left[\frac{2m_{11}}{m_{20}-m_{02}}\right],$$

where *m*_11_, *m*_20_ and *m*_02_ represent respectably the standard moment, quadratic moment according to the horizontal Cartesian axis and quadratic moment according to the vertical Cartesian axis (which is associated with the direction $\left(\frac{\pi }{2}+\varphi \right)$).

The rate of symmetry is measured through the following steps:

- making the rotation of the object following the two principal axes (thought object); and
- making the intersection between the original object and the thought object.

Finally, the exact symmetry rate of the object is deduced by taking a maximum of these four values. In this case, the symmetry rate is equal to 0.84.

**Figure 6:**
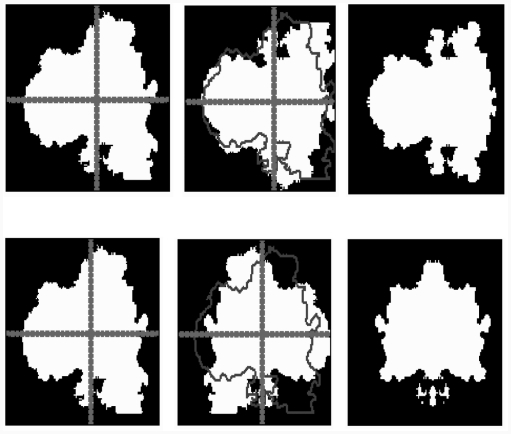
Calculating the symmetry following the two principal axes.

For this example, we find that small indentations (figure 6) lead to a low index of symmetry. In our case, the symmetry is characterized by four values; we can locate the lesions on both symmetric and asymmetric axes. This entails a number of errors relating to the asymmetry definition, which includes elements that do not translate easily into mathematical terms.

#### 4.1.2. Lengthening index

This measurement is used to describe the lengthening and the anisotropy degree of the lesion. The extension of a lesion is related to eigenvalues λ′, λ″ of the inertia matrix. The relationship between the moment of inertia around the principal axis λ′ and the moment of inertia around the secondary axis λ″ quantifies the lengthening rate [10].

(6) $$\begin{array}{l} A=\frac{\lambda^{\prime }}{\lambda^{\prime \prime }}\text{with }\lambda^{\prime }=\frac{m_{20}-m_{02}-\sqrt{(m_{20}-m_{02})+4{\left(m_{11}\right)}^{2}}}{2}\\ \lambda^{\prime \prime }=\frac{m_{20}+m_{02}+\sqrt{{\left(m_{20}-m_{02}\right)}^{2}+4{\left(m_{11}\right)}^{2}}}{2} \end{array}$$

### 4.2. Border irregularity

First, we show the irregularity of the border in order to give an overview of the edge type that can be found. We can see when the lesions are defined by clear boundaries. The agreement between doctors is strong enough to type the lesion ‘regular edges’. But, when measuring the lesion edge, it is fuzzy and less homogeneous. The number of votes (doctors) for irregular edges is also increased. Therefore, the irregularity parameter in a lesion was presented as a very important factor when evaluating a malignant lesion. In this section, we used two special features to quantify irregular edges: form parameters and fractal dimension.

#### 4.2.1. Form parameters

The edge irregularity is difficult to quantify since it depends on the precision of edge definition. The other criteria most often used to represent the ‘form irregularity’ are (figure 4(*a*)):

- the area *a* = 5946 (pixels);
- the perimeter *p* = 285 (pixels); and
- the compactness *C* defined by: $C=\frac{p^{2}}{4\pi a}=1.09$.

#### 4.2.2. Fractal dimension

Many methods exist to analyse the scale of the edge structures. Different studies have been carried out on images using fractal analysis [11]. This allows the repetition of the structure to be measured at a certain scale, and can be implemented on a grey-scale version of the image. The fractal dimension *d* is an essential parameter which is related to the *n* elements and the dilatation ratio 1/*k*. The fractal dimension is given by:

(7) $$n={\left(1/k\right)}^{-d}$$

also:

(8) $$d_{frac}=\frac{\text{log}n}{\text{log}k}.$$

In this paper, we used the box counting algorithm [12] to determine the fractal dimension. It follows the steps given below:

- the original image is divided into an image of *m* × *m*;
- the image of *m* × *m* is then divided into cells of *s* × *s*;
- the dilatation ratio is then *k* = *m/s*; then
- the number of cells (*n*) containing a portion of the edge is calculated.

(9) $$d_{frac}=\text{log}(n)/\text{log}(k).$$

**Figure 7:**
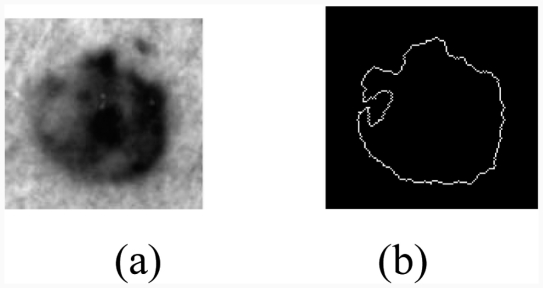
Fractal dimension calculation using the method box counting, (a) original image, (b) edge of the tumor.

When tracing log(*n*) according to log(*k*), we obtain a line passing through the origin, and its coefficients provide the fractal dimension in the picture *m* × *m* (figure 7).

### 4.3. Colour criterion

Our objective in this section is the detection of colour information contained in the lesion. It is calculated in the image frequency colour characteristics of tumours. When generating the k-means algorithm, the colour characteristics are determined by taking a sample of a few pixels in each image from the database. The k-means method is based on dividing the image grid in the RGB color space that best represent all colours present in all tumours. This method has been tested on the selected images from our skin image database. These images were collected from patients referred by the pigmented lesion in the CHUT. They were RGB colour images digitized from a hand-help video microscopy camera using a 20 times magnification lens. Each image contained 486 × 512 pixels with the special resolution of 25 μm × 33 μm. The colour content of the image will strongly depend on the photographic arrangement, e.g. lighting, flash and angles have been standardized for the images in our skin image database. Before the images could be used for the feasibility test, they were processed by two automatic pre-processing programs to extract the colour contained in the lesion. First, the skin image was checked for dark thick hairs. These hairs were removed using a software program called DullRazor [3] to reduce interference with the automated k-means program. Then the colour information was extracted automatically using the k-means algorithm. This algorithm [13] is a post-clustering technique that is widely used in image coding and pattern recognition. A sequence of iterations starts with some initial set ${\bar{C}}^{\left(0\right)}$ At each iteration *t*, all data points *c* ∈ *C* are assigned to one of the clusters ${\bar{S_{k}}}^{\left(t\right)}$ as defined in equation (10). A new centred ${\bar{C}}_{\left(k\right)}^{\left(t\right)}$ for a cluster is computed as follows:

(10) $${\bar{c_{j}}}^{\left(t+1\right)}=\frac{1}{t}\sum_{i=1}^{t}\left(c_{i}|c_{i}\in {\bar{S_{k}}}^{\left(t\right)}\right)$$

and

(11) $$\text{and }S_{k}=\{c\in C:q(c)=\bar{c_{k}}\}$$

**Figure 8:**
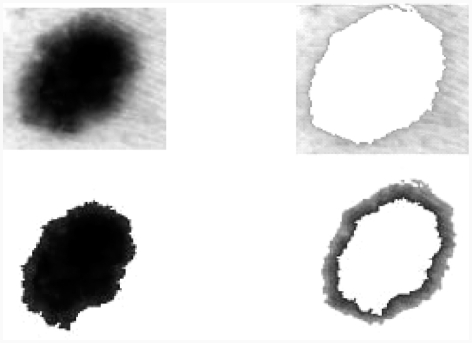
Image segmentation by k-means algorithm.

Sk : The quantization mapping defines a set of clusters equation (11). This algorithm is known to converge to a local minimum and it allows extraction of colours present in images [14]. For the test images it produced smaller average errors $\in_{q(C,I)}=\frac{1}{M}\sum_{(x,y)\in I}\left\|c_{\left(x,y\right)}-q(c_{\left(x,y\right)})\right\|$ than the median cut and variance-based pre-clustering algorithms. Unfortunately, the high cost of computation makes k-means impractical for image quantization (figure 8).

After the extraction colours presented in the lesion, we can indicate if the lesion is benign or malign.

## 5. Classification

We have seen that, in addition to the difficulty of standardizing the diagnostic criteria and the wide variability of the encountered structures, discrimination of certain types of lesion remains problematic. A system that allows analysis of tumours would be useful, especially for general practitioners who do not often observe melanomas (one case every four years on average) [2].

**Figure 9:**
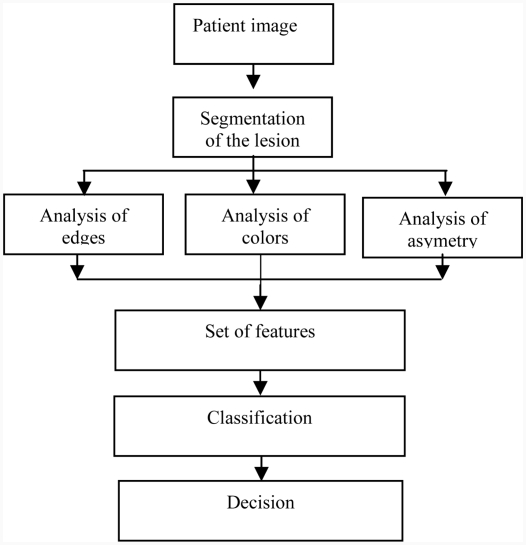
Classification algorithm of tumour skin [15].

Such a system is introduced in figure 9, which presents a general methodology based on the extraction of pertinent parameters.

The previous steps allow a set of values to be calculated that describe the tumour by a number of characteristics established by dermatologists. In order to classify the tumour as melanoma or benign, a multilayer neural network with supervised learning algorithms is used [4].

### 5.1. Multilayer neural network

**Figure 10:**
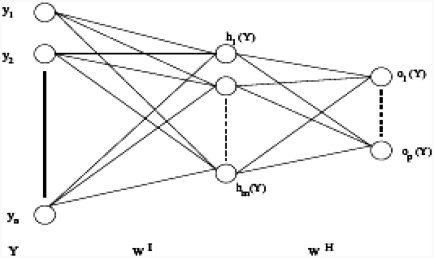
Multilayer neural network.

In the multilayer neural network the neurons are arranged by layer. The neurons of the first layer are related to external data and receive the input vector. The characteristic vector of an object is transmitted to all the neurons in the first layer of the neural network. The outputs of the neurons in this layer are then communicated to the neurons in the next layer, and so forth (figure 10). The last layer of the network is called the output layer, and the others are hidden layers [16].

(12) $$(Y=(y_{1},y_{2},\ldots ,y_{n}),$$

where *n* is the number of input attributes.

(13) $$\forall j\in \{1,2,\ldots ,m\},h_{j}\left[Y\right]=\text{tanh}\left(\sum_{i=1}^{n}w_{ji}^{I}y_{i}+w_{j0}^{I}\right)$$

where: *h* represents the activation function, $w_{ji}^{I}$ is the vector weight connecting the input *i* and layer *j*, $w_{j0}^{I}$ is the threshold of the hidden unit and *m* is the number of units in the hidden layer.

The learning algorithm of multilayer networks, known as the back-propagation algorithm, requires that the activation functions of neurons are continuous and derivable [17, 18]. In our case, the network architecture is defined by six entry units representing different attributes describing the tumours (C, A, As, *d_frac_*, C_comp_, D). The classifier differentiates between benign lesions and malignant tumours. In this case, the back-propagation algorithm minimizes squared error ɛ_*r*_ between the desired output and the input. The classification process can study and acquire experience on melanomas and benign lesions. There are a number of arbitrary parameters whose values must be defined for the network to get good performance, in particular, the number of hidden layers and the number of iterations. In our case, the errors ɛ_*r*_ are less than 0.1 and the number of iterations which assure the convergence of the network is equal to 100 iterations.

### 5.2. Results

In this application we used a database of over 180 images, which has been validated by a survey of dermatologists in the CHUT (Centre hospitalier Universitaire de Tlemcen, Algeria). Images of malignant lesions represent 40% of the overall database and therefore the benign lesions represent the remaining 60%. The used classifier allows separation of all the images into two independent sets. Therefore, the data are arranged according to a desired output calculated from previous steps which represent both cases. The size of the learning vector should be large and represent all data to ensure a good rate of classification. On the other hand, the size of the input vector must also be large to ensure good results. To assess the performance of the network, we chose to use training (T1), testing (T2) and percentiles (*k*). The obtained results are summarized in table 1 for different numbers of hidden units (*n*) so that the effect of architecture on the performance could be assessed. This table records correct detection rates, using the perceptron classifier on training set T1. Accuracy of classification on the testing set is evaluated in terms of sensitivity Sn (percentage of malignant lesions correctly classified) and specificity Sp (percentage of benign lesions correctly classified). For each couple (*k*, *n*), the network weights are initialized randomly over [−1, 1] in every execution and the final result, given in table 1, is calculated as the average over a set of 100 executions. When we compare the success within the classification rates (TCR) for the all cases studied training/testing combinations (*k*), we can conclude that correct classifications are recorded for both training and testing between 65% and 74%.

**Table 1:** Neural network diagnostic results.

*k*	*n*	Sn (%)	Sp (%)	TCP (%)
50/50	1	63.2	71.02	70.25
50/50	2	61.3	76.8	69.05
40/55	1	56.1	73.5	65.5
40/55	2	67.5	80.5	74.5

In conclusion, given the disposed database, the perceptron with one hidden layer composed of four units leads to better results with correct classification rate (TCR) of 74.5%, sensitivity (sn) of 67.5% and specificity (sp) of 80.5%. These results are comparable with the detection rates of very experienced dermatologists.

## 6. Conclusion

We have proposed an approach that allows us to test and evaluate attribute discrimination according to the studied indicators. Our objective is to determine the information referenced by dermatologists, and we were able to demonstrate the feasibility of this approach through two key steps: by validating the image database and then by creating prototypes capable of recognizing an indicator.

In this work, we studied melanoma of the skin by means of image processing techniques and classification methods. We started with a pre-processing step based on a median filter and DullRazor technique for its ability to remove the noise. Then we applied the PCA method to reduce the frequency of each colour. In the second step, the segmentation approach was used to locate the tumour and extract the edge. Then, a sequence of transformation is applied to the lesion to extract its different attributes. The final operation is to construct a classifier used for several criteria (colours, asymmetry and irregular edges), allowing the diagnosis to be evaluated.
